# Active involvement of nursing staff in reporting and grading complication‐intervention events—Protocol and results of the CAMUS Pilot Nurse Delphi Study

**DOI:** 10.1002/bco2.173

**Published:** 2022-06-15

**Authors:** Christopher Soliman, Benjamin C. Thomas, Pasqualina Santaguida, Nathan Lawrentschuk, Evie Mertens, Gianluca Giannarini, Patrick Y. Wuethrich, Michael Wu, Muhammad S. Khan, Rajesh Nair, Ramesh Thurairaja, Benjamin Challacombe, Prokar Dasgupta, Sachin Malde, Niall M. Corcoran, Philippe E. Spiess, Philip Dundee, Marc A. Furrer

**Affiliations:** ^1^ Department of Urology, Royal Melbourne Hospital University of Melbourne Parkville Victoria Australia; ^2^ Department of Health Research Methodology Evidence and Impact (HEI) McMaster University Hamilton Ontario Canada; ^3^ Unit of Urology Santa Maria della Misericordia Academic Medical Centre Udine Italy; ^4^ Department of Anaesthesiology and Pain Medicine, Inselspital, Bern University Hospital University of Bern Bern Switzerland; ^5^ Department of Urology Guy's and St. Thomas NHS Foundation Trust London UK; ^6^ Department of Urology Western Health St Albans Victoria Australia; ^7^ Department of Genito‐Urinary Oncology H. Lee Moffitt Cancer Center and Research Institute Tampa Florida USA; ^8^ Department of Urology University of Bern Bern Switzerland

**Keywords:** complication grading, complication reporting, consensus paper, Delphi method, urological surgery

## Abstract

**Objectives:**

The aim of this study is to gain experienced nursing perspective on current and future complication reporting and grading in Urology, establish the CAMUS CCI and quality control the use of the Clavien‐Dindo Classification (CDC) in nursing staff.

**Subjects and Methods:**

The 12‐part REDCap‐based Delphi survey was developed in conjunction with expert nurse, urologist and methodologist input. Certified local and international inpatient and outpatient nurses specialised in urology, perioperative nurses and urology‐specific advanced practice nurses/nurse practitioners will be included. A minimum sample size of 250 participants is targeted. The survey assesses participant demographics, nursing experience and opinion on complication reporting and the proposed CAMUS reporting recommendations; grading of intervention events using the existing CDC and the proposed CAMUS Classification; and rating various clinical scenarios. Consensus will be defined as ≥75% agreement. If consensus is not reached, subsequent Delphi rounds will be performed under Steering Committee guidance.

**Results:**

Twenty participants completed the pilot survey. Median survey completion time was 58 min (IQR 40–67). The survey revealed that 85% of nursing participants believe nurses should be involved in future complication reporting and grading but currently have poor confidence and inadequate relevant background education. Overall, 100% of participants recognise the universal demand for reporting consensus and 75% hold a preference towards the CAMUS System. Limitations include variability in nursing experience, complexity of supplemental grades and survey duration.

**Conclusion:**

The integration of experienced nursing opinion and participation in complication reporting and grading systems in a modern and evolving hospital infrastructure may facilitate the assimilation of otherwise overlooked safety data. Incorporation of focused teaching into routine nursing education will be essential to ensure quality control and stimulate awareness of complication‐related burden. This, in turn, has the potential to improve patient counselling and quality of care.

## 1 INTRODUCTION

Despite remarkable advancements in surgical care and technology, routine morbidity and mortality audits and a growing tendency to practice defensive medicine, the risk of iatrogenic and surgery related adverse events remain high.^1^ The majority of these surgical complications are deemed preventable and often receive great scrutiny due to modern safety expectations.^2^, ^3^ While complication reporting and grading, to varying extents, has been implemented into practice by surgeons for many years,^4^, ^5^, ^6^, ^7^ nursing staff are often less involved in structured complication reporting and grading. Rather, the process of incident reporting (e.g., Critical Incident Reporting System [CIRS]), which was introduced to improve patient care and expand a hospital's safety infrastructure, is routinely incorporated and documented in postoperative care.^8^, ^9^

Comprehensive complication reporting and grading in the postoperative period by all healthcare personnel (including nurses, surgeons and critical care specialists) will result in a more holistic assessment of a hospital's safety framework. In particular, a uniform classification system and database for nurses and surgeons would ensure thorough and accurate reporting and grading of complication‐intervention events.^10^, ^11^

Current and pre‐existing complication systems (e.g., Clavien‐Dindo Classification [CDC],^4^ Comprehensive Complication Index [CCI],^6^ Bern CCI,^7^, ^12^ and Common Terminology Criteria for Adverse Events [CTCAE]^13^) are inherently flawed as they only account for the severity of complication‐intervention events but fail to consider and incorporate nursing insight and perception of patient burden into the reporting of complications. As such, in addition to the original Delphi study^14^ involving urologists, anaesthetists and intensive care physicians, the Complication After Major & Minor Urological Surgery (CAMUS) Collaboration has launched a second Delphi study targeting nursing staff opinion.^15^

The aim of this study is to gain experienced nursing perspective on current and future complication reporting and grading in Urology, establish the CAMUS CCI and quality control the use of the CDC in nursing staff. Nursing involvement has the potential to improve efficiency and accuracy of complication reporting and grading in centres worldwide, ensure minor interventions are not underappreciated and may, in turn, enhance their own insight into the spectrum, and consequences, of perioperative and postoperative complications.

## 2 SUBJECTS AND METHODS

The Delphi method has been increasingly used for developing consensus guidance on best practice.^16^, ^17^, ^18^, ^19^ The primary intent of the Delphi method is to address and explore clinical areas where high‐quality evidence is limited, thereby instead reaching consensus through expert and best practice opinion. The steps involved in this process will be outlined further in the study approach (see Figure S1). In brief, (i) a group of expert participants will be questioned about the issue of interest; (ii) the process is anonymous in order to avoid social pressure and conformity to a dominant view (‘bandwagon effect’); (iii) the procedure is iterative in nature, comprising several rounds of enquiry and (iv) the design of subsequent rounds is informed by a summary of the group response of the previous round. It can be tailored to the requirements of the individual research objective, ranging from open and exploratory to standardised confirmatory approaches.^20^

The original Delphi study aimed to create a standardised and reproducible assessment of perioperative complications and overall associated morbidity by introduction of the CAMUS Reporting and Classification System (see Table S1).

In this novel nurse targeted pilot Delphi study, we aim the following (see Table 1):

1. Evaluate nursing opinion on current and future involvement and interest in complication reporting (formal/informal) and grading.
2. Have nursing participants assess the severity of various complication‐intervention events using a 0–100 scale (i.e., proposed CAMUS Grade) which will later be used to develop and validate the CAMUS CCI.
3. Quality control the use of the CDC by nursing staff, clarify areas for improvement and requirements for future involvement and assess the need for further education in nurses.
4. Gather nursing opinion on the appropriate detail and complexity required for a useable complication reporting and grading system (i.e., CAMUS supplemental grades).

**TABLE 1:** Nurse Delphi study aims

#	Aims
1	Evaluate nursing opinion on current and future involvement and interest in complication reporting (formal/informal) and grading.
2	Have nursing participants assess the severity of various complication‐intervention events using a 0–100 scale (i.e., proposed CAMUS Grade) which will later be used to develop and validate the CAMUS CCI.
3	Quality control the use of the CDC by nursing staff, clarify areas for improvement and requirements for future involvement, and assess the need for further education in nurses.
4	Gather nursing opinion on the appropriate detail and complexity required for a useable complication reporting and grading system (i.e., CAMUS supplemental grades).

### 2.1 Study approach

An outline for the nurse Delphi study approach including the method selection of nominated expert participants, invitations and reminders to participants, sample size and response rate, defining and achieving final consensus and Steering Committee selection can be seen in Table 2 (A).

**TABLE 2:** Study method

A: Study approach		
#	Section	Question
1	Delphi method	11 steps of the Delphi Study (see Figure 1a).
2	Selection of nominated expert participants	Certified local and international inpatient and outpatient nurses specialised in urology, perioperative nurses (i.e., anaesthetic nurses, recovery nurses), and urology‐specific advanced practice nurses/nurse practitioners will be invited to complete the survey. Primarily urology‐experienced perioperative nurses will be targeted (not required for inclusion).
3	Invitations and reminders to Delphi participants	Participant experts will be contacted via email (obtained via CAMUS databases and trial coordinators) and word of mouth. All participants will be invited to complete all Delphi rounds. Round 1 will be closed after 12 weeks. Reminders will be sent every 7–14 days.
4	Sample size and response rate for the Delphi Study	Minimum sample size of 250 participants will be targeted. In case of withdrawal, participants will be contacted to identify reason for withdrawal.
5	Defining and achieving final consensus	Consensus will be defined, a priori, as majority agreement (75% or greater) of participant response. If consensus is not reached then subsequent Delphi rounds (minimum of 2, maximum of 5) will be performed under guidance of the Steering Committee.
6	Steering Committee selection	Committee members will be selected primarily from our team of investigators, as well as several highly experienced specialists (nurses, urologists, critical care physicians and methodologists) in surgical reporting and grading. The selected committee will prepare and provide group feedback to participants, as well as rectify outstanding items, or add new items, if consensus is not achieved.

B: Description, content, aims and rationale of the 12 parts of the Nurse Delphi Survey		
Part	Description	Content, aims and rationale
1	Participant demographic details	Basic participant demographic information (e.g., age, location, experience) to assist in quality assurance.
2	Complication reporting and grading; now and in the future	Information regarding institutional‐driven complication reporting and grading, gauging degree of participant experience in reporting and grading, and opinions on current and potentially future nursing involvement in complication reporting & grading.
3A	Grading intervention events – CAMUS Classification	Obtain consensus on severity of a wide range of complication‐intervention events using the new CAMUS Classification.
3B	Grading intervention events – Clavien‐Dindo Classification (CDC)	Obtain consensus on severity of a wide range of complication‐intervention events using the CDC, the current standard for grading complications.
3C	Rating scenarios	Range of various scenarios requiring consensus that aim to challenge current perceptions of complication reporting, highlight pitfalls and complexities of the CDC, and finally consider potential solutions, updates, and ultimately reach consensus.
3D	CAMUS extended grade	Introduction and opinion of the new ‘CAMUS extended grade’ (see Table 1).
3E	CAMUS intra‐operative grade	Introduction and opinion of the new ‘CAMUS intra‐operative grade’ (see Table 1).
3F	CAMUS post‐operative grade	Introduction and opinion of the new ‘CAMUS post‐operative grade’ (see Table 1).
3G	CAMUS disability adjunct grade	Introduction and opinion of the new ‘CAMUS disability adjunct grade’ (see Table 1).
3H	Supplemental CAMUS grades – summative example and opinion	Provides a summative example of all the supplemental grades, as described above, to show its clinical and practical applicability.
4	Grading scenarios	Practical ‘multi‐intervention’ scenarios as a supplement to PARTS 3A‐G and aim to clarify which specific events, and the overall number of intervention events, people would consider reportable for each scenario.
5	Participant identification and consent	Requests the full name, preferred title, and professional affiliations of each participant performing the nurse Delphi survey for quality assurance and will further ensure participants are compensated for their time with PubMed listed collaborative authorship.

Individuals will be selected based on experience and presumed knowledge of surgical outcomes. Participants invited to complete the survey are local and international inpatient and outpatient nurses specialised in urology, perioperative nurses (i.e., anaesthetic nurses and recovery nurses) and urology‐specific advanced practice nurses/nurse practitioners. Our assumption is that any certified nurse will have a reasonable amount of experience in perioperative and postoperative care and can hence judge on severity of adverse surgical outcomes and complication‐intervention events during their hospital stay or mid‐ to long‐term follow‐up.

Delphi participants identified will initially be contacted using emails obtained from the CAMUS Collaboration database, Urological nursing associations and word of mouth. Participants will also be encouraged to invite their peers to participate, utilising the ‘snowball sampling’ approach to increase sample size.

All participants will be invited to complete each Delphi round, unless they withdraw at any stage. Consent will be assumed if participants complete and submit the survey. Email reminders will be sent to all primary selected participants every 14 days following survey distribution. Round 1 will close after 12 weeks.

No standard limit on when a question is considered to have reached consensus exists.^21^, ^22^ Ideally, consensus between participants over 90% would give substantial confidence that agreement is reached; however, this threshold may require additional Delphi rounds which is neither practical nor necessary. As such, in agreement with our original Delphi study,^14^ consensus will be defined as majority agreement (75% or greater) of participant response.^23^

Prior to finalising the nurse Delphi survey, a pilot survey run with 20 participants (*n* = 20 based on previous author Delphi experience) was performed in December 2021 to ensure comprehension, receive final feedback and gauge duration and functionality of the survey.^18^, ^23^ The 20 participants, all of whom met inclusion criteria, were recruited via international centres involved in the CAMUS Collaboration by personalised email, and a variety of experienced inpatient and outpatient nurses were targeted to ensure cohort diversity. The number 20 was chosen based on author experience from the original Delphi study,^14^ which yielded meaningful data and general feedback that enhanced the overall survey quality prior to its final release.

Ethics approval for this study was obtained Epworth ID: EH2021‐708, and the study was registered on ClinicalTrials.gov PRS (NCT05272592).

### 2.2 Study development and structure

The Delphi survey was created using the online REDCap software.^24^, ^25^ REDCap (Research Electronic Data Capture) is traditionally a metadata‐driven EDC software and workflow methodology for designing clinical and translational research databases and will provide a secure working platform for all data collected and distribution of subsequent Delphi rounds. It is simple for participants to complete and easy to distribute and anonymise. In addition to its intuitive interface for validated data entry, it allows automated export procedures for further data download to facilitate our statistical analysis.

The survey itself will be structured into 12 parts (1, 2, 3A, 3B, 3C, 3D, 3E, 3F, 3G, 3H, 4 and 5) and is elaborated in the survey outline (see Table 2, B). For quality control, the questionnaire was developed in conjunction with expert nurse (i.e., urology advanced practice nurses with >15 years of experience), urologist (i.e., consultant urologists with >25 years experiences and/or high‐volume urological surgeons) and methodologist input.

The outcomes from this nurse Delphi study will permit development of the CAMUS Comprehensive Complication Index (CCI). The CCI will gather physician, nurse and patient opinion and provide the ultimate appraisal of disease burden following major and minor urological surgery. This, in turn, will allow for distribution of the best possible counsel and guidance to patients as well as practising nurses and physicians.

## 3 RESULTS

All 20 participants completed the survey. Median time for completion of the survey was 58 min (IQR 40–67). Table 3 (A–F) shows the preliminary results of the pilot study.

**TABLE 3:** Preliminary results of the Nurse Delphi study pilot and amendments to the Delphi survey

A: Demographic details	
Demographic	*N* = 20
Age (year)	36.5 (31–43.5)
Gender	
Male	7 (35)
Female	13 (65)
Professional title	
Enrolled nurse (EN)	4 (20)
Registered nurse (RN)	6 (30)
Clinical nurse specialist (CNS)	2 (10)
Nurse practitioner (NP)	4 (20)
Research/study nurse	4 (20)
Main area of work	
Outpatients	10 (50)
Ward	3 (15)
Theatre	1 (5)
Recovery	3 (15)
Anaesthetics	0 (0)
ICU/HDU	3 (15)
Area of practice	
Public	14 (70)
Private	3 (15)
Public and private	3 (15)
Country	
Australia	5 (25)
US	3 (15)
UK	3 (15)
Switzerland	3 (15)
Italy	3 (15)
Germany	3 (15)
Years since completion of training	15 (11–23.25)
Main area(s) of subspecialisation	
Uro‐oncology	13 (65)
Functional urology	5 (25)
Female urology	3 (15)
Reconstructive urology	6 (30)
Andrology	3 (15)
Urolithiasis	6 (30)
Renal Transplant	6 (30)
General Urology	9 (45)
Number of patients reviewed (per day)	
None	0 (0)
1–4	11 (55)
5–9	2 (10)
10–14	7 (35)
15 or more	0 (0)

B: Experience and opinions on complication reporting^a^	
Question	*N* = 20
Complication reporting and Delphi experience	
Previous involvement in validating a reporting system	
Yes	3 (15)
No	17 (85)
Previous Delphi experience	
Yes	0 (0)
No	20 (100)
Previous position paper experience	
Yes	1 (5)
No	19 (95)
Previous experience grading complication	
Yes	5 (25)
No	15 (75)
Systems personally encountered	
CDC	16 (80)
CCI	0 (0)
Bern‐CCI	0 (0)
CTCAE	5 (25)
None	4 (20)
Reproducible systems	
CDC	8 (40)
CCI	0 (0)
Bern‐CCI	0 (0)
CTCAE	2 (10)
None	10 (50)
Complication within your institution	
Institution‐recorded prospective database	
Yes	8 (40)
No	10 (50)
I do not know	2 (10)
Single institution‐allocated individual for reporting	
Yes	0 (0)
No	18 (90)
I do not know	2 (10)
Individual responsible for reporting institution data	
Consultant	5 (25)
Fellow	13 (65)
Registrar/training resident	16 (80)
Intern/rotating medical staff	7 (35)
Nursing staff (i.e., study nurse)	7 (35)
Administrative staff	0 (0)
I do not know	4 (20)
Institution nursing involvement in informal reporting	
Yes	17 (85)
No	3 (15)
Institution nursing involvement in grading	
Yes	9 (25)
No	14 (70)
I do not know	1 (5)
Institution nursing involvement in audit preparation	
Yes	6 (30)
No	14 (70)
Institution nursing involvement in audit presentation	
Yes	5 (25)
No	15 (75)
Consensus in Urological complication reporting	
Universal demand for reporting consensus	
Yes	20 (100)
No	0 (0)
Non‐urology‐specific grading system	
Yes	16 (80)
No	0 (0)
Indifferent	4 (20)
Most important factor of new scoring system	
Reproducibility	7 (35)
Ease of use	9 (45)
Broad uptake	3 (15)
Specific urological protocol approach	1 (5)
Benefit from patient experience data	
Yes	18 (90)
No	2 (10)
Anonymised complication registry	
Yes	19 (95)
No	1 (5)
Confidential centralised audit process	
Yes	20 (100)
No	0 (0)
Surgeon obligation to provide outcome data to patients	
Yes	14 (70)
No	6 (30)
Confidence in entering codes into a complication scoring tool	
Yes	13 (65)
No	7 (35)
Nursing involvement in complication reporting & grading	
Nursing interest in reporting & grading	
Yes; reporting	7 (35)
Yes; grading	0
Yes; both	6 (30)
No	7 (35)
Should nurses be involved in reporting & grading	
Yes; reporting	3 (15)
Yes; grading	7 (35)
Yes; both	7 (35)
No	3 (15)
Specifically trained nursing involvement only	
Yes; reporting	9 (35)
Yes; grading	1 (5)
Yes; both	7 (35)
No	3 (15)
Types of nurses that should be involved	
Enrolled nurse (EN)	2 (10)
Registered nurse (RN)	6 (30)
Clinical nurse specialist (CNS)	13 (65)
Nurse practitioner (NP)	13 (65)
Clinical nurse consultant (CNC)	10 (50)
Research/study nurse	12 (60)
All nurse	3 (15)
Willingness to report & grade complications with guidance	
Yes; reporting	9 (35)
Yes; grading	0 (0)
Yes; both	8 (40)
No	3 (15)
Nursing involvement in surgical unit audits	
Yes	13 (65)
No	7 (35)
Benefit of nursing involvement to patient care	
Yes; reporting	3 (15)
Yes; grading	0 (0)
Yes; both	14 (70)
No	3 (15)
Adequate nursing training in reporting & grading	
Yes; reporting	3 (15)
Yes; grading	0 (0)
Yes; both	0 (0)
No	17 (85)
Current nursing confidence in reporting & grading	
Yes; reporting	6 (30)
Yes; grading	0 (0)
Yes; both	0 (0)
No	14 (70)
Inclusion of reporting & grading in standard nursing education	
Yes; reporting	3 (15)
Yes; grading	0 (0)
Yes; both	14 (70)
No	3 (15)
Inclusion of reporting & grading in daily nursing practice	
Yes; reporting	11 (55)
Yes; grading	0 (0)
Yes; both	5 (25)
No	4 (20)
Required collaboration between nurses & doctors in reporting & grading	
Yes; reporting	4 (20)
Yes; grading	1 (5)
Yes; both	12 (60)
No	3 (15)
Reporting & grading complications by nursing staff would negatively affect doctor‐nurse relationship	
Yes; reporting	0 (0)
Yes; grading	4 (20)
Yes; both	0 (0)
No	16 (80)
Importance of a consensus paper to clarify complication in Urology from nursing perspective	
Yes	17 (85)
No	3 (15)

C: Grading various intervention events using the proposed CAMUS Grade and CDC^b^								
Intervention‐event	CAMUS Grade* Median (IQR*)*	Clavien‐Dindo Classification (CDC)^c^ *N* (%)						
	CAMUS [0–100]	No complication	CDC1	CDC2	CDC3a	CDC3b	CDC4a	CDC4b
IDC insertion (LA)	26.5 (25–40) (6.25–13.75)	0 (0)	2 (10)	12 (60)	5 (25)	1 (5)	0 (0)	0 (0)
SPC insertion (LA)	40 (35–50)	0 (0)	1 (5)	9 (45)	9 (45)	0 (0)	1 (5)	0 (0)
SPC insertion (GA)	50 (35–75)	0 (0)	1 (5)	0 (0)	3 (15)	15 (75)	0 (0)	1 (5)
Intermittent self‐catheterisation (ISC)	31.5 (28–35)	0 (0)	3 (15)	13 (65)	4 (20)	0 (0)	0 (0)	0 (0)
Clot evacuation in theatre (GA)	50 (45–66.25)	0 (0)	0 (0)	1 (5)	1 (5)	17 (81)	0 (0)	1 (5)
Repair of bladder perforation (GA)	70 (60–85)	0 (0)	0 (0)	0 (0)	0 (0)	17 (81)	1 (5)	2 (10)
Flexible cystoscopy + dilatation (LA)	35 (30–55)	0 (0)	0 (0)	8 (40)	11 (55)	1 (5)	0 (0)	0 (0)
Stent migration requiring re‐stent (GA)	45 (39–57.5)	0 (0)	1 (5)	0 (0)	1 (5)	18 (90)	0 (0)	0 (0)
Nephrostomy insertion (LA)	35 (35–53.75)	0 (0)	1 (5)	0 (0)	18 (90)	1 (5)	0 (0)	0 (0)
Nephrostomy exchange (LA)	25 (20–30)	0 (0)	9 (45)	4 (20)	5 (25)	1 (5)	0 (0)	1 (5)
VAC dressing (GA)	50 (35–57.5)	0 (0)	0 (0)	1 (5)	0 (0)	18 (90)	1 (5)	0 (0)
Oral antibiotics	20 (12–28.75)	0 (0)	5 (25)	15 (75)	0 (0)	0 (0)	0 (0)	0 (0)
IV antibiotics	25 (22–51.25)	0 (0)	1 (5)	18 (90)	0 (0)	1 (5)	0 (0)	0 (0)
Packed red cell transfusion	23 (21–67.5)	0 (0)	0 (0)	18 (90)	0 (0)	0 (0)	1 (5)	1 (5)
HDU admission	50 (45–75)	0 (0)	0 (0)	2 (10)	1 (5)	1 (5)	16 (80)	0 (0)
ICU admission	77.5 (65–90.75)	0 (0)	0 (0)	0 (0)	0 (0)	2 (10)	16 (80)	2 (10)
Physiotherapy (pelvic floor training) for incontinence	10 (9.25–15)	1 (5)	19 (95)	0 (0)	0 (0)	0 (0)	0 (0)	0 (0)
Ultrasound‐guided aspiration of lymphocele WITHOUT drain insertion (LA)	31 (30–38.75)	0 (0)	1 (5)	7 (35)	11 (55)	1 (5)	0 (0)	0 (0)
Ultrasound‐guided aspiration of lymphocele WITH drain insertion (LA)	12.5 (10–20)	0 (0)	0 (0)	6 (30)	13 (65)	0 (0)	1 (5)	0 (0)
Sodium bicarbonate (acute)	8 (8–25)	1 (5)	17 (85)	2 (10)	0 (0)	0 (0)	0 (0)	0 (0)
Sodium bicarbonate (lifelong)	15 (12–41.5)	1 (5)	17 (85)	1 (5)	1 (5)	0 (0)	0 (0)	0 (0)
NGT insertion on ward for ileus	30 (23–35)	0 (0)	10 (50)	8 (40)	0 (0)	2 (10)	0 (0)	0 (0)
Total parental nutrition (via PICC) (TPN)	31 (28–61.25)	0 (0)	1 (5)	14 (70)	4 (20)	0 (0)	0 (0)	1 (5)
Exploratory laparotomy/laparoscopy WITHOUT stoma (GA)	80 (70–83.75)	0 (0)	0 (0)	0 (0)	0 (0)	18 (90)	1 (5)	1 (5)
Exploratory laparotomy/laparoscopy WITH stoma (GA)	88 (85–93)	0 (0)	0 (0)	0 (0)	0 (0)	17 (85)	1 (5)	2 (10)

D: Opinions on complication reporting.	
Question	*N* = 20
**Complication reporting and Delphi experience**	
Reporting both complications and interventions (vs. interventions alone)	
Yes, both	17 (85)
No, intervention‐based	3 (15)
No, complication‐based	0 (0)
Parameters required for reporting	
POD complication	20 (100)
POD each intervention	20 (100)
POD ONLY most severe intervention	15 (75)
Grading all interventions	15 (75)
Complication description	14 (70)
Intervention description	13 (65)
Description & code of complication	15 (75)
Description & code of intervention	16 (80)

E: Opinion on various complication reporting scenarios.	
Scenarios	*N* = 20
Specific scenario regarding ‘death’	
Reporting of death	
CDC5/CAMUS10	10 (50)
Grade X	10 (50)
Specific scenario regarding ‘unrelated’ post‐operative events	
Reporting unrelated events occurring POD <90	
Yes	7 (35)
No	13 (65)
Specific scenarios regarding complication‐intervention events beyond 90 days after surgery	
Reporting directly related complications >90 days postoperative	
Yes	20 (100)
No	0 (0)
Duration of reporting	
6 months	1 (5)
12 months	2 (10)
18 months	0 (0)
24 months	1 (5)
3 years	1 (5)
5 years	0 (0)
10 years	3 (15)
Until end of follow up/death	12 (60)
General scenarios	
Reporting readmissions	
Yes	20 (100)
No	0 (0)
Reporting rehabilitation due to global decondition	
Yes	15 (75)
No	5 (25)
Reporting rehabilitation due to specific complication	
Yes	18 (90)
No	2 (10)
Reporting need for hospital in the home (HITH)	
Yes	12 (60)
No	8 (40)
Reporting routine post‐operative medications	
Yes	2 (10)
No	18 (90)
Specific scenarios regarding recurrent interventions	
Reporting recurrent interventions individually – physiotherapy session	
Yes	1 (5)
No	19 (95)
Reporting recurrent interventions individually – VAC change	
Yes	8 (40)
No	12 (60)
Grading scenarios	
*Reporting the following intervention events*.	
Hospital‐acquired pneumonia requiring (1) oxygen, (2) chest physio, (3) IV antibiotics, (4) ICU admission, (5) intubation & ventilatory support, (6) daily Chest X‐Rays, (7) bronchoscopy, (8) tracheostomy.	
No intervention	0 (0)
Intervention 1	2 (10)
Intervention 2	6 (29)
Intervention 3	17 (81)
Intervention 4	20 (95)
Intervention 5	19 (90)
Intervention 6	4 (19)
Intervention 7	20 (95)
Intervention 8	20 (95)

F: Opinion on the proposed CAMUS supplemental grades.	
Scenarios	*N* = 20
CAMUS extended grade	
Incorporation of the extended grade	
Yes	20 (100)
No	0 (0)
Including number of interventions in e‐grade	
Yes	20 (100)
No	0 (0)
CAMUS intra‐operative grade	
Reporting of intra‐operative complications	
Yes	20 (100)
No	0 (0)
Reporting intra‐op complication requiring additional unplanned interventions during same anaesthesia	
(Disagree) 0	0 (0)
1	0 (0)
2	0 (0)
3	0 (0)
4	0 (0)
5	0 (0)
6	3 (15)
7	4 (20)
8	0 (0)
9	7 (35)
(Agree) 10	6 (30)
Reporting intra‐op complications requiring no post‐op interventions	
(Disagree) 0	0 (0)
1	0 (0)
2	0 (0)
3	0 (0)
4	0 (0)
5	0 (0)
6	4 (20)
7	2 (10)
8	4 (20)
9	3 (15)
(Agree) 10	7 (35)
Incorporation of the intra‐operative grade	
Yes	20 (100)
No	0 (0)
CAMUS post‐operative grade	
Reporting complications without any interventions	
Yes	17 (85)
No	3 (15)
Reporting of asymptomatic complication without intervention	
Within CDC/CAMUS	1 (5)
Supplemental method co‐reported with CDC/CAMUS	2 (10)
Separate post‐op reporting system	4 (20)
Does not require reporting	13 (65)
Reporting of symptomatic complication without intervention	
Within CDC/CAMUS	1 (5)
Supplemental method co‐reported with CDC/CAMUS	9 (45)
Separate post‐op reporting system	10 (50)
Does not require reporting	0 (0)
CAMUS disability‐adjunct grade	
Reporting frequent, minor complications	
Yes	16 (80)
No	4 (20)
Defining frequency	
Daily	1 (5)
Every 2–3 days	4 (20)
Weekly	8 (40)
Fortnightly	6 (30)
Monthly	3 (15)
6‐weekly	0 (0)
3 monthly	0 (0)
Other	0 (0)
Reporting frequent, major complications	
Yes	20 (100)
No	0 (0)
Overall opinion (CAMUS vs. CDC)	
Classification system preference	
CAMUS Classification	3 (15)
CAMUS Classification + supplemental grade(s)	1 (75)
Clavien‐Dindo Classification (CDC)	0 (0)
CDC + supplemental CAMUS grade(s)	2 (10)

*Note*: Data are reported as median (interquartile range) or frequency (%).

Abbreviations: CCI, Comprehensive Complication Index; CDC, Clavien‐Dindo Classification; CTCAE, Common Terminology Criteria for Adverse Events; LA, local anaesthesia; GA, general anaesthesia; IDC, indwelling catheter; SPC, suprapubic catheter; IV, intravenous; HDU, high‐dependency unit; ICU, intensive care unit; NGT, nasogastric tube.

^a^ Data are reported as median (interquartile range) or frequency (%).

^b^ Data are reported as median (interquartile range) or frequency (%).

^c^ Answers of all 20 Delphi participants included.

The survey showed that 85% of nursing staff report experience with informal complication reporting while only 25% of nurses have experience in grading complications, and only 25–30% are currently involved in surgical unit audit preparation and presentations.

Additionally, although nursing participants report a modest 65% interest in reporting and/or grading complications, 85% believe nurses should be involved in future complication reporting and/or grading and surgical audits. Furthermore, these nurses should be specialised nurses and nurses who have received targeted training.

Moreover, survey data revealed poor confidence in ability to perform the task and significant inadequacy in relevant background nursing education surrounding complications (only 15% and 0% report adequate training in reporting and grading, respectively). Approximately 85% believe that nursing education on reporting and/or grading is essential and that nursing involvement in reporting and/or grading will benefit patient care and improve patient outcomes.

Finally, overall, 100% of nurse participants recognise the universal demand for reporting consensus and believe that ‘ease of use’ and ‘reproducibility’ are the most important factors for a novel nursing involved scoring system. With these goals in mind, the final opinion of all pilot participants revealed a 75% preference towards the CAMUS Classification and supplemental grades, as compared to the CDC.

Several amendments were made following feedback and the survey for Round 1 of the nurse Delphi study was then finalised for distribution. Table 4 highlights the main amendments to the survey following pilot review and feedback.

**TABLE 4:** Amendments to Delphi survey following pilot study results and feedback

#	Amendments
1	Removal of baseline characteristic questions inappropriate for nursing staff.
2	Rephrasing of multiple questions to improve clarity.
3	Duration (minutes) taken to complete each section.
4	Consideration of formal vs. informal complication reporting

## 4 DISCUSSION

A lack of global consensus on reporting and grading complications hampers the conclusive assessment of urological procedures and the ability to compare longitudinal outcome data. Therefore, a urology‐specific reporting and classification system (i.e., CAMUS System) is necessary; however, reporting and grading tools are useful only if appropriately validated with all involved parties (i.e., nurses, surgeons and critical care physicians) and then widely accepted and integrated by the entire urological community.

For several reasons, the inclusion of nursing staff is essential in successfully creating such a tool (see Table 5).

1. *Nurses are principally positioned to identify complications early before harm occurs and are knowledgeable regarding the normal and abnormal postoperative patient course.*

**TABLE 5:** Importance of including nursing staff opinion in creating the CAMUS CCI

#	Rationale
1	Nurses are principally positioned to identify complications early before harm occurs and are knowledgeable regarding the normal and abnormal postoperative patient course.
2	Minor interventions, and assessment of morbidity, may be underappreciated without the contribution of nurses to complication‐intervention event reporting.
3	Prospective data collection may improve efficiency and accuracy of complication reporting and grading if integrated into routine nursing documentation.
4	Advanced practice nurses are heavily involved in pre‐ and post‐operative care of complex urological procedures and should be a utilised asset in the remodelling of complication reporting and grading.
5	Nursing involvement in the reporting and grading of adverse events may enhance their own insight into the spectrum, and consequences, of peri‐ and postoperative complications.

Beyond training, nurses are often streamlined into specialty and subspeciality based areas of care (e.g., urology) and become highly experienced in providing appropriate postoperative management. Nurses ensure execution of postoperative interventions and care plans, monitor and assess patients closely for clinical deterioration and perform effective record keeping and documentation to support the provision of safe, high‐quality patient care.^26^

Effective management of postoperative complications requires early recognition, efficient communication with relevant team members and prompt treatment. Nurses are primely placed at the forefront of healthcare systems with significant responsibility to recognise any concerning deviation from the expected postoperative course. A combination of consistent patient interaction and healthcare systems safeguards (i.e., baseline and routine perioperative observation monitoring) facilitates recognition of signs of haemorrhage, shock or sepsis and ensure timely involvement of medical staff. Therefore, the appreciation of complication‐intervention events by nursing staff is not only of great value but is a requisite that must be incorporated into the CCI.

- *Minor interventions, and assessment of morbidity, may be underappreciated without the contribution of nurses to complication‐intervention event reporting.*

The frequency of nursing patient interaction, as compared to surgeon patient, significantly increases the likelihood that a minor complication will be recognised. Nurses perform a considerable number of independent intervention‐events (bedside or outpatient) without surgeon involvement (i.e., wound dressing, VAC changes and administration of non‐critical medications). In addition, nurses are more likely to recognise complication associated disease burden. Patients may indicate to nurses if certain symptoms are bothersome with greater detail and if additional therapy is required. This appreciated morbidity should be reflected in the development of the CCI.

- *Prospective data collection may improve efficiency and accuracy of complication reporting and grading if integrated into routine nursing documentation.*

The emphasis on nursing record‐keeping provides ample opportunity to integrate complication data collection. A database may be created to allow easy and immediate recording of complications in real time. At present, complication reporting and grading data are often collected retrospectively by surgeons, captured predominately through review of nursing and clinician documentation. Thus, in contrast, real time integration would considerably increase speed, efficiency and accuracy of complication reporting and grading. Furthermore, there may be potential in the future to combine complication reporting and classification systems with notifiable incident reporting (i.e., CIRS).

- *Advanced practice nurses are heavily involved in preoperative and postoperative care of complex urological procedures and should be a utilised asset in the remodelling of complication reporting and grading.*

Many complex oncological and non‐oncological urological conditions often require intensive mid‐ to long‐term follow‐up. Modern urological units employ advanced practice nurses or nurse practitioners to undertake subspecialised, time intensive tasks (e.g., perioperative prostatectomy counselling and self‐catheterisation education after orthotopic bladder substitution).

The incorporation of these experienced clinical nurses into urological units creates robust longitudinal nurse–patient relationships which ensure high patient satisfaction. Nurses are then privy to a patients' psychological mindset and potentially cognisant of any long‐term complication associated burden. Information that is again invaluable to the CCI development.

- *Nursing involvement in the reporting and grading of adverse events may enhance their own insight into the spectrum, and consequences, of perioperative and postoperative complications.*

Frequent utilisation of a complication reporting and classification system may present nursing staff with a valuable learning opportunity for the identification and remediation of factors that contribute to a complication before further harm occurs.^27^ Nursing participation in this Delphi survey may, if successful, ignite this future potential for routine nursing involvement and stimulate the recognition of perioperative and postoperative complications on both an academic and clinical level.

This may significantly assist in reducing overall patient morbidity and mortality.^28^

However, success and practicability of this reporting and classification system's use by nursing staff are dependent on several factors.^29^ First, nurses must be aware of the complication reporting and classification system and understand the logistics of its use. Second, nurses must be confident in recognising, reporting and grading complications in the best interest of the patient and surgeon.^30^ Finally, nurses must accept complication‐intervention event reporting and grading as a non‐punitive part of everyday practice. Preventing any form of blame culture is of utmost importance to avoid barriers and maximise information gathering.^3^

Depending on the type and severity of a complication‐intervention event, the likelihood of these being reported varies. Vincent et al.^29^ and Evans et al.^30^ noted that of the perceived barriers to reporting and grading, no discrete aetiology was identified above all others, confirming the view that this issue is multifactorial.

In general, complication‐intervention events perceived as innocuous are less frequently reported compared to events that result in significant morbidity. Additionally, nursing staff may be reluctant to report complications whereby human error is clearly at fault due to fear of retribution or in contrast may not recognise a complication in a situation in which an individual may not directly attribute responsibility, such as a postoperative delirium. This interpretation may also be influenced by the overall view that some events are considered more traditional ‘complications’ compared to others.^31^ Moreover, accurate and detailed reporting of complication‐intervention events is both resource and time intensive in nature.

Given the potential disparity and risk of inter‐rater variability between nursing staff, clear definitions and instructions are crucial to appropriately guide which complication‐interventions events meet criteria for reporting and grading. These concerns will be addressed and facilitated by use of an anonymous database with a data dictionary.

Furthermore, studies have revealed that nursing staff are confident in utilising incident reporting systems^27^, ^30^ which are currently considered operational, easily accessible and well accepted across many hospitals. However, although incident reporting systems have positively transformed attitudes towards safety and error,^9^ they are not equivocal to structured complication reporting and thus are not utilised in morbidity and mortality audits.^28^ In addition, study outcomes demonstrate that nurses report incidents more frequently than surgeons, likely related to unit expectations and familiarity of incident reporting systems. As such, although specialised nursing training may be required for quality control, this apparent lower threshold for reporting by nursing staff may suggest nurses are more likely to uptake and routinely utilise reporting and classification systems.^27^, ^29^, ^30^

**FIGURE 1:**
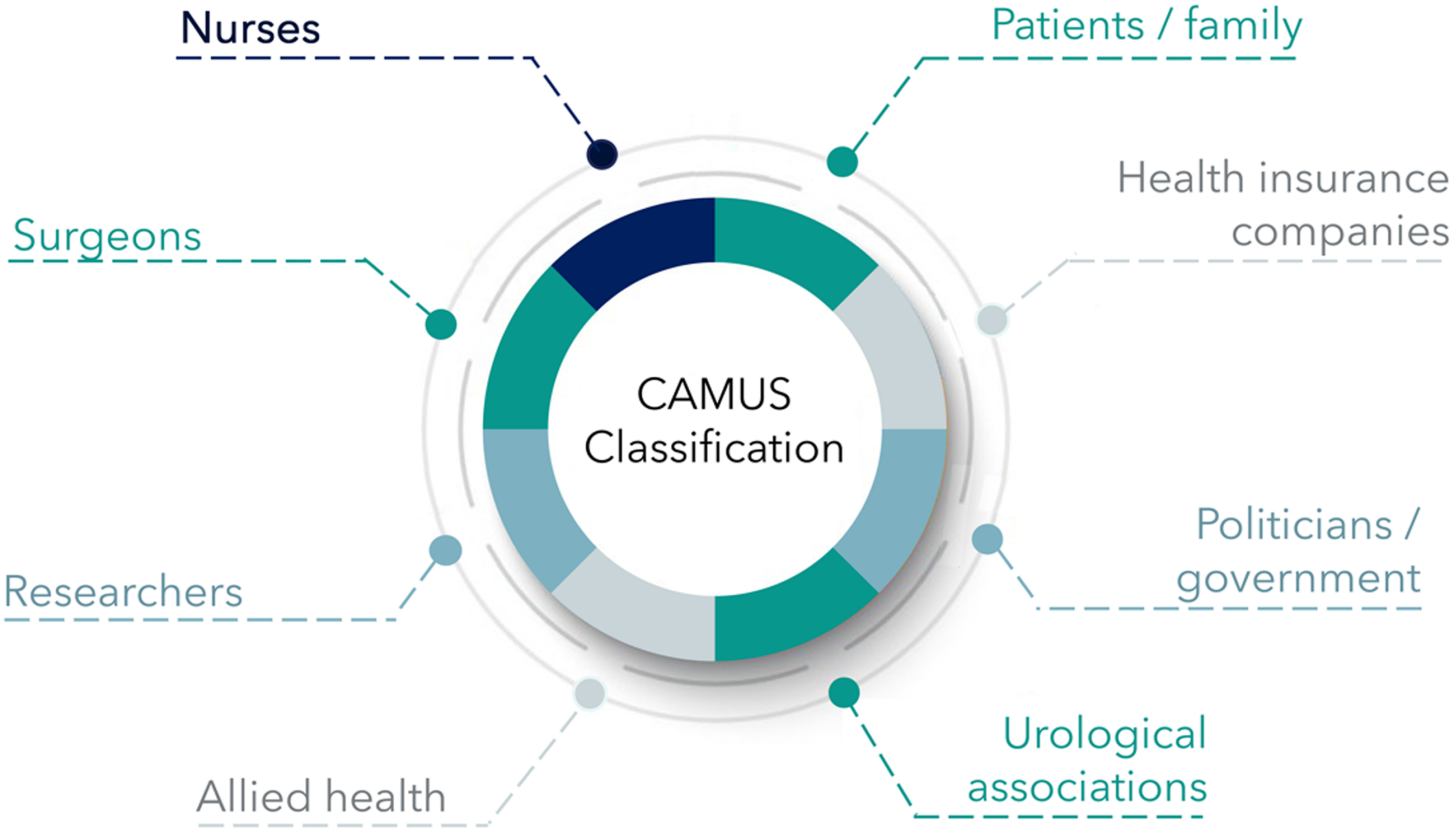
Stakeholders that benefit from a standardised complication reporting system. If the CAMUS Reporting and Classification System can be implemented worldwide, after calibration, it has potential to create reliable guidelines and recommendations, and provide benefit to all potential stakeholders (i.e., nursing staff, surgeons, units, hospitals, patients, family members/next of kin, researchers, health insurance companies, politicians and urological organisations)

The novel CAMUS System has several strengths (see Table S2) and potential implications (see Table S3). Once successfully validated by nursing staff, it may improve the accuracy, understanding and standardisation of complication reporting and grading worldwide and better reflect patient burden and quality of surgical care.^32^ Moreover, it may provide benefit to all potential stakeholders (i.e., nurses, surgeons, units, hospitals, patients, family members/next of kin, researchers, health insurance companies, politicians and urological organisations) (see Figure 1).

This Delphi study and proposed CAMUS System has several limitations including (i) variability in nursing experience, (ii) complexity of supplemental CAMUS grades, (iii) dependency on honesty, (iv) time‐consuming nature of comprehensive and accurate complication reporting, (v) risk of poor data due to participant fatigue and withdrawal (despite compensatory authorship being offered) and (vi) lack of randomisation in the nurse Delphi survey questionnaire.

However, to the best of our knowledge, although nurses have previously been involved in the formulation of complication grading tools, this is the first ever complication reporting system developed with a consideration for nursing staff insight and opinion. This insight will provide an added dimension to the understanding of patient burden.

## 5 CONCLUSION

The integration of experienced nursing opinion and participation in complication reporting and grading systems in a modern and evolving hospital infrastructure may facilitate the assimilation of otherwise overlooked safety data. This unique input may result in more consistent, higher quality reporting. Of note, incorporation of focused teaching into routine nursing education will be essential to ensure quality control and stimulate awareness and appreciation of the burden related to perioperative and postoperative complications. This, in turn, has the potential to improve patient counselling and quality of care.

## CONFLICT OF INTEREST

All authors declare no conflict of interest.

## AUTHOR CONTRIBUTIONS

The study concept and design, analysis and interpretation of data, and drafting of the manuscript was performed by Soliman and Furrer. The acquisition of data and statistical analysis was performed individually by Soliman. Supervision and the obtaining of funding was performed individually by Furrer. Administrative, technical, and material support was performed individually by Nair. Critical revision of the manuscript of important intellectual content was performed by all authors.

## Supporting information


**Figure S1.** The 11 steps of the Delphi method.
The primary intent of the Delphi method is to address and explore clinical areas where high‐quality evidence is limited, thereby instead reaching consensus through expert and best practice opinion. The 11 steps of the Delphi method listed will provide global consensus on complication reporting and grading after urological surgery.

**Table S1.** Overall CAMUS study aims
Table 2: Strengths of the CAMUS System
Table 3: Potential implications in clinical practice and research
