# Supplementary material for: Active involvement of nursing staff in reporting and grading complication‐intervention events—Protocol and results of the CAMUS Pilot Nurse Delphi Study
Source: BJUI Compass. 2022 Jun 15;3(6):466–83. doi: 10.1002/bco2.173 (PMC9579890; doi:10.1002/bco2.173)
Supplement: Supplementary file 1 — Figure S1. The 11 steps of the Delphi method. The primary intent of the Delphi method is to address and explore clinical areas where high‐quality evidence is limited, thereby instead reaching consensus through expert and best practice opinion. The 11 steps of the Delphi method listed will provide global consensus on complication reporting and grading after urological surgery. [file BCO2-3-466-s002.docx]

Supplementary Figure

**Figure 1:**

**The 11 steps of the Delphi method.**

*The primary intent of the Delphi method is to address and explore clinical areas where high-quality evidence is limited, thereby instead reaching consensus through expert and best practice opinion. The 11 steps of the Delphi method listed will provide global consensus on complication reporting and grading after urological surgery.*
