# Supplementary material for: Active involvement of nursing staff in reporting and grading complication‐intervention events—Protocol and results of the CAMUS Pilot Nurse Delphi Study
Source: BJUI Compass. 2022 Jun 15;3(6):466–83. doi: 10.1002/bco2.173 (PMC9579890; doi:10.1002/bco2.173)
Supplement: Supplementary file 2 — Table S1. Overall CAMUS study aims Table 2: Strengths of the CAMUS System Table 3: Potential implications in clinical practice and research [file BCO2-3-466-s001.docx]

Supplementary Tables

**Table 1: Overall CAMUS study aims**

| # | Aim |
| --- | --- |
| 1 | To create a uniform language in complication reporting with comprehensive criteria on how to report complication-intervention events. |
| 2 | To propose and achieve consensus of all five different CAMUS Classification grades (i.e., CAMUS grade, CAMUS extended grade, CAMUS intra-operative grade, CAMUS post-operative grade, CAMUS disability adjunct grade). |
| 3 | To propose an updated revision of the CDC specifically customised for urological surgery based on the pre-existing classification. |
| 4 | To propose an augmentation of the CDC using the CAMUS supplemental grades (i.e., extended grade, intra-operative grade, post-operative grade, and disability adjunct grade). |
| 5 | To create a basis for later development of the CAMUS CCI by using a 0-100 numerical rating scale (NRS) to grade intervention events. |
| 6 | To validate the CAMUS Reporting and Classification System in experienced nursing staff. |
| 7 | To globally validate the above outlined Delphi consensus. |

**Table 2: Strengths of the CAMUS System**

| # | *Strengths* |
| --- | --- |
| 1 | CAMUS Classification is not purely defined by grade of anaesthesia. |
| 2 | Ten-grade CAMUS scoring system (vs. seven-grade CDC scoring system) allows for greater differentiation between grading of complication-intervention events. |
| 3 | Addresses the many difficulties of complication vs. intervention-event based grading. |
| 4 | Allows for longitudinal assessment of complications (beyond 90 days). |
| 5 | Comprehensive and accurate reporting to allow for true standardisation and comparability of complications and surgical care for all stakeholders (i.e., surgeons, units, hospitals, patients, family members/next of kin (NOK), researchers, nurses, health insurance companies, politicians, urological organisations) (see Figure 1e). |
| 6 | Allows for the assessment of intra-operative complications and complication-intervention events, combined with postoperative complication-intervention events, using a single all-inclusive system (i.e., CAMUS Classification) vs. multiple separate systems for intra- (e.g., ClassIntra or EAUiaiC) and postoperative (e.g., CDC) complication-intervention events. |
| 7 | Generalisability and applicability to all other surgical specialities (e.g., consensus grading of many common complications, follow-up beyond 90 days for a complex procedure, abandonment of a procedure). |
| 8 | Extended grade has ease and usability but provides more comprehensive assessment of post-operative complication-intervention events. |
| 9 | Supplemental CAMUS grades (i-, p-, and DA- grades) will allow for reporting of intra-operative complications, post-operative complications and frequent minor complications that have never been appropriately represented with the CDC or CCI. |
| 10 | Provides a potential solution for reporting complications which occur that are obviously unrelated to a surgical procedure or that are expected sequalae of a procedure. |
| 11 | Better overall representation of patient morbidity associated with any given intra-operative or post-operative complication, regardless of whether interventions are required. |
| 12 | Improved pre-operative patient counselling and informed consent of potential operative risks and complications (short-term and long-term). |
| 13 | Provides surgeons and juniors with an improved understanding and awareness of expected post-operative course and complication-intervention events that may be required, ultimately improving doctor- and unit- preparedness and patient quality of care. |
| 14 | Improve clarity and provide a clear recommendation on how to report complications (see Table 4b) and which complications are reportable (i.e., using a uniform language and coding dictionary to classify all potential complications). |
| 15 | Improved surgeon motivation to report complication-intervention events given the new standardisation and guidance. |
| 16 | Inter-surgeon and inter-hospital comparison will stimulate competition between surgeons to better their surgical outcomes and ultimately improve patient quality of care. |
| 17 | Potential for the CAMUS Classification to be introduced in the form of an online grading & reporting tool and registry for surgeons to record and store all complications on a single worldwide, anonymous database. |
| 18 | Potential for the above (#16) to be de-anonymised, should political/government bodies desire, and audit complications for quality control or publish complication-registry results to the public to allow patients to compare centres and choose their preferred surgeon or facility. This, in turn, would again improve competition and quality of surgical care. |

**Table 3: Potential implications in clinical practice and research**

| # | *Implications* |
| --- | --- |
| 1 | Stimulate competition between urologists and centres internationally. |
| 2 | Improved surgical quality and unit efficiency. |
| 3 | Provides a better understanding of intra- and post-operative morbidity. |
| 4 | Offer transparency for patient counselling regarding potential surgical morbidity. |
| 5 | Improve accuracy and quality of patient consent. |
| 6 | Creation of an online grading & reporting tool and registry for surgeons to record and store all complications on a single worldwide, anonymous database. |
| 7 | Potential for the above (#6) to be de-anonymised, should government bodies desire, and audit complications for quality control or publish complication-registry results to the public to allow patients to compare centres and choose their preferred surgeon or facility. |
| 8 | Creates an opportunistic window for a wide variety of clinical research, including prospective randomised and non-randomised trials. |
